# Post-translational modification crosstalk in acute pancreatitis: linking acinar stress to innate immune amplification and tissue repair

**DOI:** 10.3389/fimmu.2026.1935553

**Published:** 2026-09-16

**Authors:** Mengwei Chen, Peng An, Shenglong Jing, Longlong Wu, Rong Wang

**Affiliations:** 1 Department of Gastroenterology of Shanxi Provincial People’s Hospital, Shanxi Medical University, Taiyuan, China; 2 Department of Colorectal and Anal Surgery of Shanxi Provincial People’s Hospital, Shanxi Medical University, Taiyuan, China

**Keywords:** acute pancreatitis, immunometabolism, innate immunity, macrophage polarization, neutrophil extracellular traps, post-translational modifications, sterile inflammation

## Abstract

Acute pancreatitis (AP) is a sterile inflammatory disease initiated by acinar cell stress and amplified through innate immune, vascular, and inter-organ responses, yet effective disease-modifying therapies remain unavailable. Although premature digestive enzyme activation, organelle stress, sterile inflammation, and microvascular failure are well-recognized features of AP, the mechanisms linking early acinar stress to coordinated injury across epithelial, immune, stromal, and vascular compartments remain insufficiently integrated. Emerging evidence suggests that post-translational modifications (PTMs) constitute a dynamic regulatory layer through which metabolic stress is converted into damage-associated molecular pattern release, innate immune activation, inflammatory cell death, tissue repair, and disease progression. This review examines how metabolic stress remodels lactylation, acetylation, phosphorylation, ubiquitination, and related PTMs in AP and how their functional convergence and selected PTM interactions shape DAMP sensing, inflammasome activation, macrophage reprogramming, neutrophil recruitment and extracellular trap formation, cytokine amplification, mitochondrial dysfunction, apoptosis, pyroptosis, ferroptosis, microvascular barrier failure, and systemic complications. We propose an integrated framework linking acinar stress to PTM remodeling, innate immune amplification, and inflammatory resolution. Across acinar, ductal, immune, stellate, and endothelial compartments, PTMs regulate protein stability, subcellular localization, transcriptional programs, organelle quality control, and intercellular signaling. Selected PTM pathways exhibit stage- and cell-dependent effects; for example, lactate-associated lactylation may exacerbate early ferroptotic injury while promoting reparative macrophage polarization during recovery. This framework positions PTM remodeling as a context-dependent immunometabolic control system while recognizing that direct molecular PTM–PTM crosstalk remains incompletely demonstrated in AP.

## Introduction

Acute pancreatitis (AP) remains one of the leading gastrointestinal causes of hospitalization, and its incidence continues to rise worldwide (1–3). Although most cases are self-limited, nearly one-fifth progress to local or systemic complications, including pancreatic necrosis, persistent organ failure, and prolonged intensive care requirements (1, 4). Supportive management has improved and care has become more standardized, yet no mechanism-based therapy has consistently prevented the transition from early pancreatic injury to systemic deterioration (5). This therapeutic gap reflects a broader conceptual problem: AP is still often described as a collection of separate pathogenic events, whereas clinical progression is driven by their temporal coupling and multicellular amplification (1).

The classical framework of AP centers on premature digestive enzyme activation in acinar cells (6). This remains important, but it no longer explains the full disease process. Acinar injury is now understood to involve calcium overload, endoplasmic reticulum stress, mitochondrial depolarization, ATP depletion, defective autophagy, and inflammatory cell death (6–8). These processes do not simply coexist; they reinforce one another and determine whether an early secretory stress response is contained or progresses to necroinflammation (6–8). Clinical guidelines likewise emphasize that early organ dysfunction is closely linked to systemic inflammatory responses, endothelial injury, and vascular leakage, indicating that disease severity cannot be understood solely through enzyme activation (4, 5, 8). The clinically relevant question is therefore not only how AP starts, but how local acinar stress is translated into tissue-wide and ultimately systemic injury (6–8).

At the same time, the field has become richer but also more fragmented. Single-cell and spatially resolved studies increasingly define AP as a multicellular disease characterized by dynamic state transitions rather than a purely acinar catastrophe (9– 11). Pancreas-focused transcriptomic studies have shown coordinated remodeling across acinar, immune, and other pancreatic compartments during both injury and recovery, including acinar-to-ductal metaplasia, early neutrophil influx, dendritic cell expansion, and marked macrophage reprogramming driven by resident-cell activation and monocyte recruitment (9, 10). Additional work has shown that inflammatory amplification is sustained by early neutrophil and macrophage activation, whereas endothelial hyperpermeability is both a consequence of injury and an active driver of extra-pancreatic organ damage (12). These observations indicate that AP progression is governed by intercellular crosstalk across epithelial, immune, stromal, and vascular niches (9–12).

However, defining the participating cell types does not by itself explain how diverse stress signals are integrated into coherent pathological outcomes. We propose that post-translational modifications (PTMs) provide this missing regulatory layer. PTMs can rapidly reprogram protein activity, stability, localization, complex assembly, organelle quality control, and transcriptional output without requiring new protein synthesis. More importantly, they couple metabolic flux to inflammatory and stress signaling. This concept is particularly relevant in AP, where calcium toxicity, oxidative stress, mitochondrial dysfunction, disrupted proteostasis, and metabolic rewiring arise early and evolve rapidly. In this context, phosphorylation, acetylation, ubiquitination, lactylation, and related modifications should not be viewed as isolated biochemical details, but as a regulatory language through which metabolic stress is translated into inflammatory amplification, cell-death decisions, barrier failure, and repair responses. Here, we distinguish strict molecular PTM crosstalk—where one modification directly alters the installation, removal, occupancy, or functional consequence of another modification on the same or a functionally coupled substrate—from broader pathway convergence, in which distinct PTM-regulated axes influence a shared inflammatory or stress phenotype. Because direct AP-specific evidence for the former remains limited, this review explicitly distinguishes direct PTM evidence from PTM-associated pathway evidence and mechanistic extrapolation from non-AP settings (13, 14).

Recent studies in AP support this framework. Lactate-associated signaling provides a clear example: a recent study linked lactate and H3K18 lactylation to aggravated pancreatic injury through modulation of NCOA4-mediated ferroptosis (15). Yet the same axis may support recovery, as lactate has also been shown to promote reparative macrophage polarization and pancreatic repair in a stage-dependent context (16). Similar complexity is emerging for other PTMs. Acetylation has moved beyond descriptive epigenetics, with EP300-dependent acetylation of HSF1 stabilizing a protective HSF1-PRKN mitophagy axis in acinar cells (17). On the ubiquitin side, recent studies have implicated deubiquitinases in both acinar and immune compartments, including USP7 in autophagy- and macrophage-associated inflammatory reprogramming and USP25 as a negative regulator of macrophage TBK1-NF-κB signaling (18). These findings reposition PTMs from peripheral modifiers to candidate organizers of AP pathobiology (19).

Accordingly, this review examines how metabolic stress remodels interconnected PTM networks and how these networks convert acinar injury into damage-associated molecular pattern release, innate immune activation, inflammatory cell death, endothelial dysfunction, and systemic inflammation. Particular attention is given to macrophage and neutrophil reprogramming, inflammasome-associated signaling, immunovascular injury, and the stage-dependent transition from inflammatory amplification to tissue repair. By integrating these processes, we treat PTM remodeling and selected PTM interactions as a context-dependent immunometabolic control system rather than as a collection of isolated biochemical modifications. However, direct AP-specific evidence remains limited for several PTM classes, and mechanisms inferred from other inflammatory diseases should be interpreted cautiously. Accordingly, the evidence is organized along the sequence acinar metabolic/organelle stress → PTM remodeling → innate immune and vascular amplification → inflammatory resolution or maladaptive repair, with cell type, disease stage, and evidentiary strength considered at each step.

## Literature search and evidence-integration approach

This narrative review was updated using structured searches of PubMed/MEDLINE and Europe PMC from database inception through September 1, 2026. The core strategy combined “acute pancreatitis” or “severe acute pancreatitis” with post-translational modification terms and the major classes discussed here, including lactylation, acetylation, phosphorylation, ubiquitination/deubiquitination, O-GlcNAcylation, and SUMOylation; targeted searches were also performed for AP-relevant regulators and substrates. English-language peer-reviewed or formally accepted studies were considered. AP-specific original studies were prioritized for mechanistic claims, whereas reviews were used mainly for background or evidence synthesis. Non-AP studies were retained only when they supplied mechanistic context unavailable in AP and were labeled extrapolative. Evidence was integrated at three levels: direct AP PTM evidence, in which a modification was measured or manipulated and linked to a functional outcome; AP-associated evidence, in which a PTM-regulatory enzyme, pathway, whole-tissue omics signal, or circulating marker was implicated without demonstration of a specific endogenous PTM event; and extrapolated non-AP evidence. Cell attribution was considered direct only when supported by isolated cells, cell-specific manipulation, or cell-resolved data. Whole-pancreas omics, systemic pharmacology, blood lactate, and circulating proteins were not interpreted as cell-specific PTM evidence without direct validation. Reference metadata and citation-to-claim alignment were audited before revision.

## Metabolism-coupled PTM remodeling in acute pancreatitis

In AP, metabolic stress functions as an early organizing force rather than a secondary consequence of tissue injury and determines whether acinar stress remains locally contained or progresses to self-amplifying inflammation, organelle failure, and tissue necrosis (20). Contemporary models of AP increasingly converge on the view that pathological calcium signaling, mitochondrial depolarization, ATP depletion, redox imbalance, defective autophagic flux, and endoplasmic reticulum (ER) stress arise early and reinforce one another (21, 22). In this context, PTMs extend beyond peripheral modifications superimposed on already activated pathways and represent a principal mechanism through which metabolic disequilibrium is translated into sustained changes in protein function and cell fate (20).

This perspective is important because most PTMs are metabolically regulated. Their installation and removal depend on substrate availability, cofactor abundance, enzyme localization, and the cellular stress state. In AP, mitochondrial dysfunction reduces ATP production and perturbs the NAD+/NADH balance; ER stress disrupts proteostasis and calcium homeostasis; impaired autophagy prevents the clearance of damaged organelles and protein aggregates; and oxidative stress reshapes thiol chemistry and stress signaling (21, 22). Overall, these changes create a biochemical environment in which phosphorylation, acetylation, ubiquitination, lactylation, and other modifications are remodeled in parallel rather than in isolation (23). The consequence is not simply an increase in PTMs, but a shift in signaling logic: proteins undergo changes in stability, trafficking, transcriptional activity, organelle targeting, and interactions with inflammatory complexes (24).

A useful way to conceptualize this process is to ask how metabolic stress reshapes the decision architecture of the acinar cell. Calcium overload and mitochondrial permeability transition pore (mPTP) opening reduce bioenergetic reserve, rendering ATP-dependent homeostatic programs more difficult to sustain. At the same time, reactive oxygen species (ROS) accumulation and defective mitophagy increase the persistence of damaged mitochondria, thereby amplifying inflammasome signaling and promoting more destructive cell death programs (25). In parallel, ER stress and lysosomal-autophagic dysfunction impair protein quality control, such that stress-responsive PTM enzymes act in a setting in which both substrate proteins and the organelles that host them are already abnormal (21–23). Therefore, PTM remodeling in AP is best understood not as a late epigenetic refinement, but as an early stress-adaptive process that may become maladaptive when bioenergetic failure is prolonged (26, 27).

Recent primary studies support this model by demonstrating that metabolism and PTM regulation are already experimentally linked in AP (17–19). In pancreatic acinar cells, EP300-dependent acetylation stabilizes HSF1, preserves PRKN-mediated mitophagy, reduces ROS production, and restrains NLRP3 activation; when this acetylation axis collapses, mitochondrial quality control fails and injury worsens (17). In the immune compartment, USP7 has been shown to regulate macrophage polarization in severe AP through PKM2-dependent metabolic reprogramming, thereby linking a deubiquitinating enzyme to glycolytic state and inflammatory phenotype (18). These studies are important not because they explain all PTM behavior in AP, but because they directly illustrate the underlying principle: metabolic stress actively shapes PTM remodeling rather than simply accompanying it (28).

For this reason, the most informative question is no longer which PTM is present in AP, but which metabolic inputs bias PTM networks toward injury or recovery (28, 29). Lactate, acetyl-CoA, NAD+, ROS, and ubiquitin-dependent proteostasis define the biochemical conditions under which PTM-regulated axes can converge (28, 29). The lactate-lactylation axis is particularly informative because it links glycolysis with macrophage plasticity, ferroptosis, and tissue repair (15, 16).

## Lactylation

Lactylation is the PTM most directly linking metabolic flux to gene regulation. First identified in 2019 as a lactate-derived lysine modification on histones, it provided a framework for understanding how glycolytic output can be translated into transcriptional change (30). Since then, the field has expanded beyond histones, and both histone and non-histone lactylation are now considered biologically relevant. Beyond p300/CBP, HDAC1–3 have been identified as delactylases, whereas AARS1/AARS2 and HBO1 have emerged as candidate lactyltransferases (31–34). Recent chemical studies further indicate that lysine L-lactylation is the dominant glycolysis-induced lactylation isomer, although the underlying chemistry and enzymatic logic remain incompletely resolved (35). This uncertainty is particularly important in AP, as it cautions against overinterpretation of lactylation-related signals.

In AP, lactylation is especially compelling because lactate biology is already clinically and experimentally relevant. Elevated blood lactate levels and impaired lactate clearance are associated with persistent organ failure and with short- and long-term mortality, indicating that lactate is more than a nonspecific marker of critical illness in this disease (36–38). Recovery models also indicate enhanced glycolysis and increased lactate levels during injury, providing a plausible context for lactylation, particularly in inflammatory and reparative cell populations undergoing rapid metabolic reprogramming. Still, elevated lactate alone should not be equated with pathogenic lactylation; the key question is how lactate is interpreted by different cell types at different stages of disease.

The most important recent insight is that lactylation in AP is not unidirectional. A recent mechanistic study showed that lactate and H3K18 lactylation aggravate experimental AP, at least partly through NCOA4-mediated ferroptosis (15). Lactate levels increased in AP and correlated positively with pancreatic injury and inflammation. CUT&Tag, RNA sequencing, ChIP-qPCR, and promoter analyses further supported a model in which H3K18la increased NCOA4 transcription, thereby promoting ferroptotic injury (15). Conceptually, this finding links a metabolite-associated chromatin mark to a defined cell death program rather than only to broad inflammatory readouts. It also aligns with the broader view that metabolic dysregulation can drive AP toward oxidative lipid damage and ferroptosis.

Equally important, another recent study reached a different conclusion during recovery. In a caerulein-induced recovery model, Jiang and colleagues found that exogenous lactate promoted pancreatic repair, whereas Pfkfb3 deficiency lowered lactate levels and delayed recovery. Mechanistically, lactate shifted macrophages from a pro-inflammatory toward a reparative phenotype, increased lactylation, enhanced repair-associated gene expression, and suppressed JAK2–STAT1 signaling through GPR132-dependent mechanisms (16). Rather than contradicting the ferroptosis-oriented findings, this study highlights a central feature of lactylation biology in AP: its effects are stage-dependent and cell-context-dependent. Early in disease, the lactate–lactylation axis may intensify acinar injury and ferroptosis; during resolution, the same axis may promote macrophage reprogramming and tissue repair.

Earlier work outside AP showed that the TLR signaling adaptor BCAP promotes the transition from inflammatory to reparative macrophages by enhancing histone lactylation (39). Although that study did not examine pancreatitis, it supports the broader concept that lactylation can favor inflammatory resolution in a context-dependent manner. The available evidence argues against a simplistic view in which lactylation is either pathogenic or protective. Lactylation may instead function as a context-sensitive signal whose effects depend on glycolytic burden, inflammatory timing, and cellular identity.

For this review, lactylation is valuable both as an additional PTM and as a prototype for the broader argument developed here. It shows how a simple metabolite can be converted into a PTM signal that alters chromatin state, macrophage plasticity, ferroptosis susceptibility, and tissue repair (15, 16). Therapeutic intervention therefore cannot target lactylation in the abstract. Future studies should determine when, where, and in which cell populations the lactate-lactylation axis is maladaptive and when it supports repair. This distinction will determine whether lactylation can serve as a biomarker or therapeutic target and reinforces the need for stage-specific PTM-directed strategies. Thus, lactylation may function as a stage-dependent immunometabolic switch that links early acinar ferroptosis to subsequent macrophage-mediated inflammatory resolution.

## Acetylation

Acetylation is among the most metabolically sensitive PTMs and, in AP, one of the easiest to oversimplify. It is often introduced mainly as an epigenetic mechanism; however, emerging evidence in AP indicates a broader role in stress adaptation, spanning chromatin regulation, mitochondrial quality control, proteostasis, and inflammatory threshold setting. An integrative pancreatic atlas of AP and severe acute pancreatitis (SAP) identified 1,677 acetylated proteins and demonstrated substantial remodeling of the acetylome across normal, AP, and SAP states, indicating that acetylation is a systems-level feature of disease progression rather than a niche epigenetic add-on (20).

The strongest recent mechanistic evidence places acetylation upstream of mitochondrial quality control in acinar cells. Zhu and colleagues showed that EP300 protects against AP by acetylating HSF1, thereby stabilizing HSF1 and promoting PRKN-mediated mitophagy in pancreatic acinar cells. When this EP300–HSF1 axis is disrupted, HSF1 undergoes proteasomal degradation, mitophagic capacity declines, and pancreatic injury worsens (17). This finding demonstrates that acetylation in AP extends beyond transcriptional fine-tuning and can determine whether damaged mitochondria are cleared or allowed to persist as amplifiers of ROS production, inflammasome activation, and susceptibility to cell death. In this setting, acetylation acts less as a static chromatin mark than as a switch governing whether stress responses remain adaptive.

At the same time, AP evidence argues against a simplistic model in which increased acetylation is uniformly harmful and reduced acetylation uniformly beneficial. The deacetylation arm can also be protective, depending on substrate and context. FGF21 alleviates experimental AP by upregulating SIRT1 in acinar cells, restoring impaired autophagy, and facilitating removal of damaged organelles (40). PINK1/PARK2-dependent mitophagy also suppresses NLRP3 inflammasome activation and pancreatic injury (41). In addition, SIRT4 alleviates severe AP by regulating the HIF-1α/HO-1 axis and inhibiting ferroptosis, further linking acetylation-related metabolic control to cell-death decisions under oxidative stress (42). These findings indicate that acetylation biology in AP is governed by substrate selectivity and subcellular context: EP300-dependent acetylation of some proteins is protective, whereas sirtuin-dependent deacetylation of others can be equally protective.

This context dependence becomes clearer beyond a single enzyme or compartment. Experimental studies have shown that HDAC activity contributes to pancreatitis severity: HDAC inhibition reduced trypsin activation, inflammation, and tissue injury; class I HDAC blockade limited leukocyte recruitment and acinar-toductal metaplasia; SAHA acted through an HDAC5/SLIT2/AKT/β-catenin axis; and HDAC1 promoted AP through ATF4-associated signaling (43–46). More recently, betulinic acid was reported to alleviate AP by promoting SIRT1–PINK1-mediated mitophagy, with SIRT1 knockdown counteracting both its anti-inflammatory and promitophagic effects (47). These observations indicate that acetylation is a pharmacologically tractable regulatory system rather than only a descriptive biomarker layer. However, this does not imply that broad acetylation-targeted therapy will be uniformly effective; therapeutic value will depend on defining which acetylation-dependent nodes are adaptive and should be preserved and which become maladaptive and should be interrupted. By regulating mitophagy, mitochondrial DAMP release, and inflammasome thresholds, acetylation-related circuits indirectly determine the intensity of innate immune activation in AP.

## Phosphorylation

Among the PTMs implicated in AP, phosphorylation is the most rapid and pervasive. It serves as an early signaling language through which acinar cells interpret calcium overload, ATP depletion, receptor activation, and innate immune sensing. Yet phosphorylation is also the PTM most often presented in a flat, pathway-by-pathway manner, which obscures its broader significance. In AP, phosphorylation extends beyond activating discrete cascades such as MAPK, PI3K/AKT, NF-κB, or JAK/STAT and organizes rapid state transitions from secretory stress to organelle failure, from local injury to inflammatory amplification, and from potentially reversible damage to regulated cell death. Large-scale phosphoproteomic data support this view: the integrative pancreatic atlas of AP and SAP identified 3,130 phosphorylated proteins, with altered phosphorylation involving RIPK1, FASN, SLC3A2, PCBP2, SLC38A2, STAT3, VAV2, and AKT1 (20).

A useful way to interpret phosphorylation in AP is to focus on decision nodes rather than pathway lists. One such node is AMPK, which senses energetic stress and can redirect acinar cells away from catastrophic injury. In obese mouse models of severe AP, AMPK activation ameliorated disease by suppressing pancreatic acinar cell necroptosis (48). These findings reposition phosphorylation as more than a simple inflammatory trigger: selected phosphorylation events preserve bioenergetic adaptation and quality-control pathways when the acinar cell is approaching the point of no return. This is precisely why a blanket anti-kinase strategy is unlikely to succeed in AP, as the same modification class contains both destructive amplifiers and stress-buffering restraints.

A second major phosphorylation-defined decision point lies in regulated necrosis. Inhibition of RIPK1-dependent regulated acinar cell necrosis and attenuation of the RIPK1/RIPK3/MLKL pathway protect against experimental AP, supporting an injurious role for selected necroptotic signaling circuits (49, 50). However, a separate study reported that RIPK3- and MLKL-mediated necroptosis may protect against exacerbation of AP in certain settings and cautioned against indiscriminate use of necroptosis inhibitors (51). MLKL signaling also shapes macrophage polarization through CXCL10 (52), while circulating RIPK3 and MLKL have been associated with AP severity (53). In parallel, PSD-95-mediated regulation of p38 MAPK illustrates that selected phosphorylation circuits can exert protective effects (54). These apparently divergent findings suggest that phosphorylation-dependent death signaling in AP is highly dependent on model, disease stage, and cellular compartment.

Phosphorylation also shapes immune amplification and regulated necrosis in experimental AP (19, 48, 51). In macrophages, Usp25 deficiency exacerbates AP by upregulating TBK1–NF-κB signaling, leading to greater pancreatic and lung injury, increased inflammatory cell infiltration, and more pronounced systemic inflammatory responses (19). Once immune phosphorylation hubs such as TBK1 are released from restraint, they can convert local pancreatic stress into broader cytokine amplification and extra-pancreatic injury. The consequences of phosphorylation-controlled death signaling vary with cell type and experimental context. Consequently, the central translational challenge is to determine which kinase-centered circuits govern irreversible progression across cell types.

## Ubiquitination

In AP, ubiquitination functions as a selective regulatory system linking proteostasis to inflammatory intensity, autophagy, and stress adaptation. USP7 illustrates the compartment-specific nature of this regulation: it promotes inflammatory metabolic reprogramming in macrophages through PKM2 deubiquitination (18), while a separate study showed that USP7 aggravates AP by enhancing ATF4-mediated autophagy in acinar cells (55). JOSD2 promotes AP by removing K63-linked polyubiquitin chains from PCNA and enhancing DNA-damage-associated signaling in acinar cells (56). USP25 further illustrates why this field cannot be interpreted in binary terms: Usp25 deficiency exacerbates AP through macrophage TBK1–NF-κB signaling (19), whereas USP25 upregulation has been implicated in acinar-cell GSDMD-mediated pyroptosis and STAT3-associated epithelial and barrier injury (57, 58). Extracellular SQSTM1 links ubiquitin-selective autophagy machinery to ferroptotic injury in AP (59). Deubiquitinating enzymes therefore act as context-dependent checkpoints that determine protein stability, macrophage polarization, autophagy, and the persistence of pancreatic necroinflammation.

## Other post-translational modifications

This section should remain selective. Among the non-core PTMs, O-GlcNAcylation currently has the clearest direct evidence in AP: pancreas-specific reduction of OGT mitigates caerulein-induced pancreatic inflammation (60). Protein modification and degradation are central regulators of ferroptosis (61). Related AP studies show that AMPK–SIRT1 signaling restores autophagy and restrains inflammation, whereas baicalin suppresses phosphorylated-MLKL oligomerization and necroptosis (62, 63). Direct AP evidence further includes OGT-mediated NF-κB activation (64), OGT-mediated O-GlcNAcylation of ATF3 and activation of the ACSL1 ferroptotic program (65), and PIAS1-dependent Foxa2 SUMOylation with repression of FTO transcription (66) (**Figure 1**). By contrast, direct evidence in AP for methylation, succinylation, palmitoylation, or UFMylation remains limited; these modifications are better regarded as emerging candidates than established drivers (**Table 1**).

**Figure 1:**
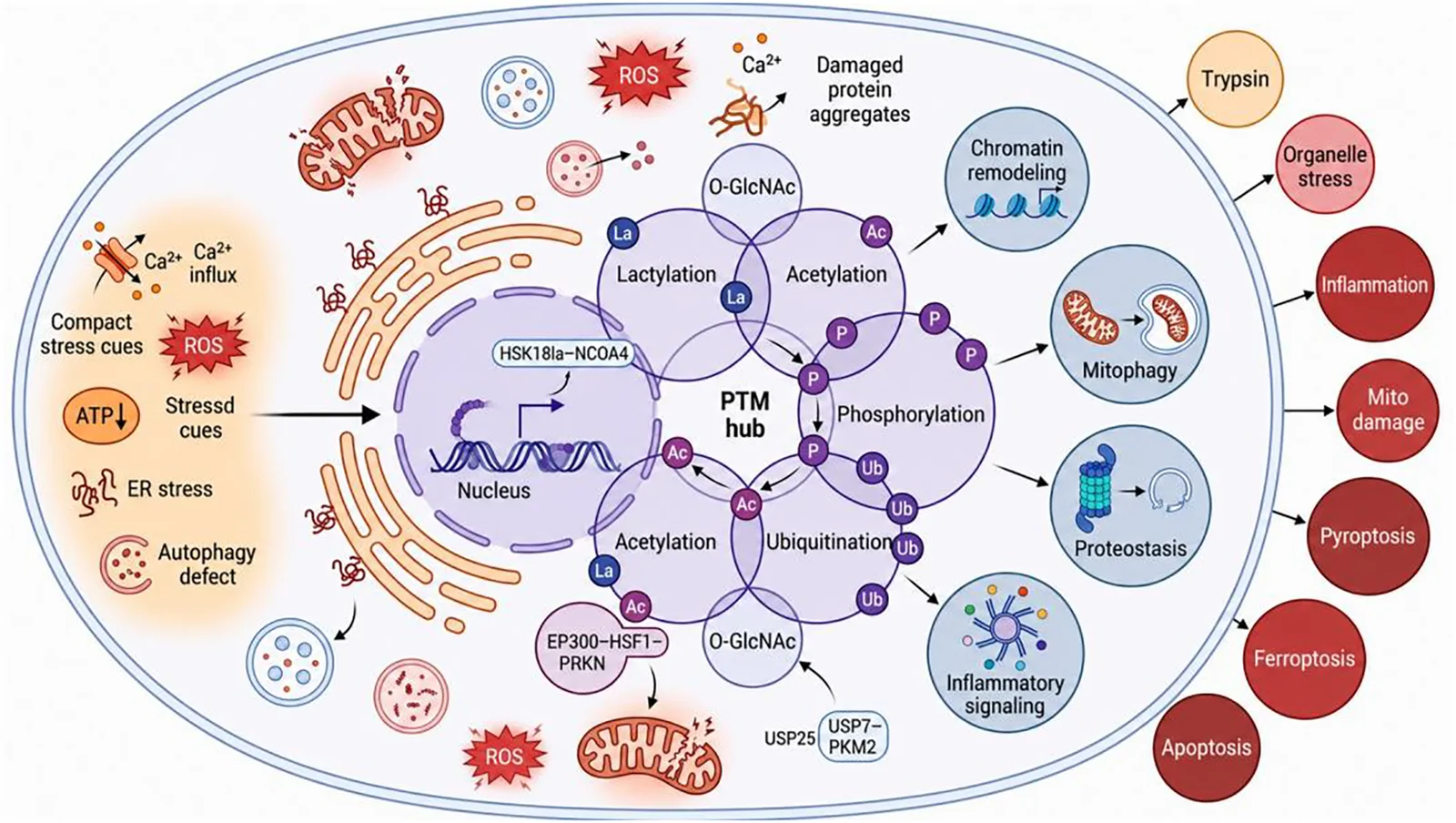
Intracellular PTM networks linking acinar stress to inflammatory signaling in acute pancreatitis. Metabolic stress in pancreatic acinar cells remodels an interconnected post-translational modification (PTM) network that converts early intracellular injury into pathological decisions in acute pancreatitis. Calcium overload, oxidative stress, ATP depletion, endoplasmic reticulum stress, and autophagy impairment converge on a spatially integrated PTM hub composed of lactylation, acetylation, phosphorylation, ubiquitination, and related modifiers. Rather than acting as isolated pathways, these PTMs coordinately regulate chromatin remodeling, mitophagy, proteostasis, and inflammatory signaling, thereby shaping downstream outputs including premature trypsin activation, organelle stress, mitochondrial damage, inflammation, pyroptosis, ferroptosis, and apoptosis. Representative regulatory axes include H3K18la–NCOA4, EP300–HSF1–PRKN, USP7–PKM2, and USP25–TBK1/NF-κB, supporting the concept that metabolic stress is translated into acinar cell fate decisions through a PTM-regulated intracellular control map. Mitochondrial DAMP release and inflammasome activation provide a major interface between acinar PTM remodeling and innate immune amplification. Representative axes denote PTM-regulated mechanisms reported in AP; the schematic does not imply that every link represents direct molecular crosstalk between distinct PTMs or that all nodes have equivalent evidentiary strength.

**Table 1:** Core PTM pathways and their immune-inflammatory consequences in acute pancreatitis.

PTM	PTM enzyme/associated regulator	Representative axis in AP	Immune-inflammatory consequence	Evidence status	Reference
Lactylation	p300/CBP; HDAC1–3; SIRT2/3	H3K18la–NCOA4; lactate–GPR132	Promotes ferroptotic injury in early AP but may support reparative macrophage polarization during recovery	A (H3K18la); A/B (stage-dependent recovery axis)	(15, 16)
Acetylation	EP300; SIRT1; SIRT4; HDACs	EP300–HSF1–PRKN; SIRT1–PINK1; SIRT4–HIF-1α/HO-1	Regulates mitophagy, autophagy, oxidative stress, and ferroptosis susceptibility	A (EP300–HSF1); B (SIRT/HDAC-associated axes without a defined substrate)	(17, 42, 47)
Phosphorylation	AMPK; TBK1; AKT; RIPK3/MLKL	AMPK–SIRT1; TBK1–NF-κB; RIPK3/MLKL	Controls rapid stress signaling, inflammatory amplification, autophagy, and regulated necrosis	B unless a specific phosphorylation event is directly quantified or perturbed	(19, 48, 51)
Ubiquitination	USP7; USP25; PRKN/PARK2; JOSD2	USP7–PKM2; USP25–TBK1/NF-κB; PINK1/PARK2	Regulates protein stability, macrophage polarization, mitophagy, and inflammatory intensity	A (USP7–PKM2); B (USP25 pathway regulation)	(18, 19, 59)
O-GlcNAcylation	OGT; OGA	OGT–ATF3–ACSL1	Aggravates severe AP by enhancing ferroptosis-associated signaling	A for measured O-GlcNAcylation; substrate/site specificity strongest for ATF3	(60, 64, 65)
SUMOylation	PIAS1; Foxa2	PIAS1–Foxa2–FTO	Supports epithelial stress adaptation and may limit injury progression	A (single preclinical AP study); C for broader contextual evidence	(66, 67)

Evidence status: A, direct AP PTM evidence (the specific PTM was measured/manipulated and functionally linked; cell attribution is direct only when cell-specific or cell-resolved); B, AP-associated pathway, omics, or enzyme evidence without a direct endogenous substrate/site or cell-specific validation; C, extrapolated/contextual evidence from non-AP settings.

## PTM-governed cellular crosstalk and immune amplification in acute pancreatitis

Cell-specific interpretation in this section follows the evidence hierarchy defined above; when the underlying evidence is derived from whole tissue, systemic intervention, or non-AP models, the mechanism is presented as compartment-associated rather than directly cell-specific.

Acinar PTM remodeling acquires immunological significance when it alters DAMP release, cytokine production, and the recruitment or functional polarization of innate immune cells. Macrophages and neutrophils subsequently amplify pancreatic injury through metabolic reprogramming, inflammasome activation, extracellular trap formation, and cytokine release, while endothelial, platelet, stromal, and regulatory immune compartments determine whether inflammation becomes systemic or resolves. The following section therefore examines AP as a PTM-linked multicellular inflammatory network rather than as an exclusively acinar-cell disorder.

## Acinar cells

Acinar cells remain the initiating site of injury in AP, but they should not be portrayed simply as passive victims of premature enzyme activation. IL-22BP has been shown to modulate acute injury while delaying subsequent tissue recovery in chronic pancreatitis (68). Integrated single-cell and bulk transcriptomic analyses have also identified ER-stress-associated acinar damage programs in AP (69). Using a pancreas-optimized transcriptomic approach, Aney and colleagues captured acinar cells across phases of injury and recovery and defined a gene program that quantifies deviation from normal acinar identity during acinar-to-ductal metaplasia (ADM) (9). Baldan et al. further showed that SOX4 restrains excessive acinar dedifferentiation and supports appropriate specification of acinar-derived metaplastic states (10). Additional mechanistic studies indicate that karyopherin α2 promotes NF-κB-dependent inflammatory signaling in acinar cells (70), whereas ER stress can drive PERK-dependent, caspase-1-mediated acinar pyroptosis (71). These findings position acinar cells as dynamic decision-makers that initiate injury, transmit danger signals to immune and stromal compartments, and influence whether tissue enters a reparative or self-perpetuating inflammatory trajectory.

## Ductal cells

Ductal cells are not innocent bystanders downstream of acinar injury. Their physiological function—secreting bicarbonate-rich fluid to maintain luminal alkalinity, flush enzymes, and preserve ductal patency—positions them at a strategic checkpoint between local acinar stress and broader exocrine failure (72–74). Alcohol can reduce CFTR abundance and function and thereby promote pancreatitis (72), while AP-associated CFTR mislocalization can be partially corrected by VX-661 plus VX-770 (73). Restoring CFTR function with Orkambi likewise reduces the severity of alcohol-induced AP (74). Single-cell mapping has revealed that the ductal tree comprises multiple populations with potential roles in regeneration and exocrine pathogenesis (75), and rare CFTR variants are associated with impaired CFTR function in a subset of patients with pancreatitis (76). Recent experimental work adds an important mechanistic dimension: MGST1 protects pancreatic ductal cells from inflammatory injury by restraining the ACSL4/GPX4 ferroptotic axis (77). In AP, acinar stress and ductal dysfunction therefore form a reciprocal circuit in which impaired ductal defense and secretion can exacerbate protease retention, oxidative damage, and epithelial injury.

## Macrophages

Macrophages extend beyond secondary responders to acinar injury and act as major interpreters of pancreatic stress. Phenotypic switching between inflammatory and repair-associated states helps orchestrate injury, resolution, and regeneration after AP (78), while recent work has revealed substantial diversity among circulating monocytes and pancreatic macrophages during pancreatitis and recovery (79). NLRP3 inflammasome signaling contributes to the balance between systemic inflammatory response syndrome and compensatory anti-inflammatory response syndrome (80). Within this heterogeneous compartment, USP7 promotes a pro-inflammatory macrophage state through PKM2 deubiquitination and metabolic reprogramming (18), whereas lactate promotes reparative polarization and pancreatic regeneration during recovery (16). Macrophages can also release extracellular traps that modulate compensatory anti-inflammatory responses through IL-33/ST2 signaling in severe AP (81). Macrophage PTM remodeling therefore represents a principal interface between local metabolic stress, systemic cytokine amplification, and inflammatory resolution.

## Neutrophils

Neutrophils are prominent first-wave innate effectors in severe AP, and neutrophil extracellular traps (NETs) connect local pancreatic injury to systemic damage (82–84). Circulating citrullinated histone H3 has shown diagnostic and prognostic potential in septic patients with AP (82), while P-selectin can promote NET formation through the PSGL-1/Syk/Ca2+/PAD4 pathway and aggravate pancreatic injury (83). A single-cell-atlas-inspired study further identified heterogeneous neutrophil states and showed that restricting neutrophil ROS can provide therapeutic benefit (84). Thus, neutrophils help convert transient epithelial injury into a self-reinforcing inflammatory circuit by promoting cell death, recruiting additional myeloid cells, and propagating damage beyond the pancreas.

## T lymphocytes and other immune cells

T lymphocytes appear later than neutrophils and macrophages in AP but contribute to the balance between inflammatory persistence and resolution. Galectin-3 deletion ameliorates experimental AP while increasing IL-10-producing Foxp3+ regulatory T cells (Tregs), supporting a protective regulatory program (85). Conversely, activated Tregs may facilitate duodenal bacterial translocation into necrotic pancreatic areas in severe AP, illustrating context-dependent adverse effects (86). Human single-cell RNA sequencing has revealed broad alterations in circulating immune-cell populations during severe AP (87), including a B-cell/plasma-cell-associated signature predictive of disease severity (88). At barrier sites, IL-22 attenuates AP-associated intestinal mucosal injury through STAT3 activation (89), while dendritic cells have been shown to promote pancreatic viability in experimental AP (90). These observations indicate that adaptive and antigen-presenting immune compartments can either support tissue protection or contribute to dysregulated host responses depending on disease context.

## Pancreatic stellate cells

Pancreatic stellate cells (PSCs) are context-dependent participants in pancreatic injury and repair. In a lineage-tracing study, pancreatic collagen-expressing fibroblasts increased during AP, correlated with histologic severity, and localized around acini, ducts, and vessels; depletion of these cells aggravated inflammation, indicating that some PSC-associated fibroblast populations can be protective (91). Activationassociated downregulation of TRPA1 can also attenuate intracellular Ca2+ signaling and protect stellate cells against alcohol-metabolite-induced death (92).

## Endothelial cells, platelets, and the microcirculation

Microvascular failure in AP is an active driver of disease progression rather than a passive consequence of inflammation. Endothelial activation, coagulation disturbance, and microcirculatory dysfunction contribute to edema, hypoperfusion, and extra-pancreatic injury. Clinical studies have identified the endothelial activation and stress index, circulating endothelial microparticles, soluble thrombomodulin, and imbalance of the vWF–ADAMTS13 axis as markers of severe disease and persistent organ failure (93–95). Glycocalyx disruption is likewise increasingly viewed as a central component of SAP-associated multiple organ dysfunction (96). At the mechanistic level, CIRP directly increases endothelial permeability through SRC activation and disruption of VE-cadherin/β-catenin junctions (12), whereas pancreatic trypsin can activate human platelets through PAR4, linking protease spillover to immunothrombotic microvascular injury (97).

## Other cells

This category should remain selective. Single-cell analysis of AP-associated intestinal injury identified activated mast cells and mast cell-derived CCL5 as drivers of early gut dysfunction and inflammatory-cell recruitment (98). Endocrine cells merit inclusion because endocrine–exocrine signaling is bidirectional and involves cytokines, paracrine factors, and the immune microenvironment (99). In AP, islet cells are unlikely to initiate injury, but they may contribute to metabolic instability and help explain why an acute exocrine insult can produce long-term endocrine consequences (**Figure 2**). These underappreciated compartments expand AP from a localized pancreatic lesion to a network disease involving distant barrier sites and intra-pancreatic endocrine communication (**Table 2**).

**Figure 2:**
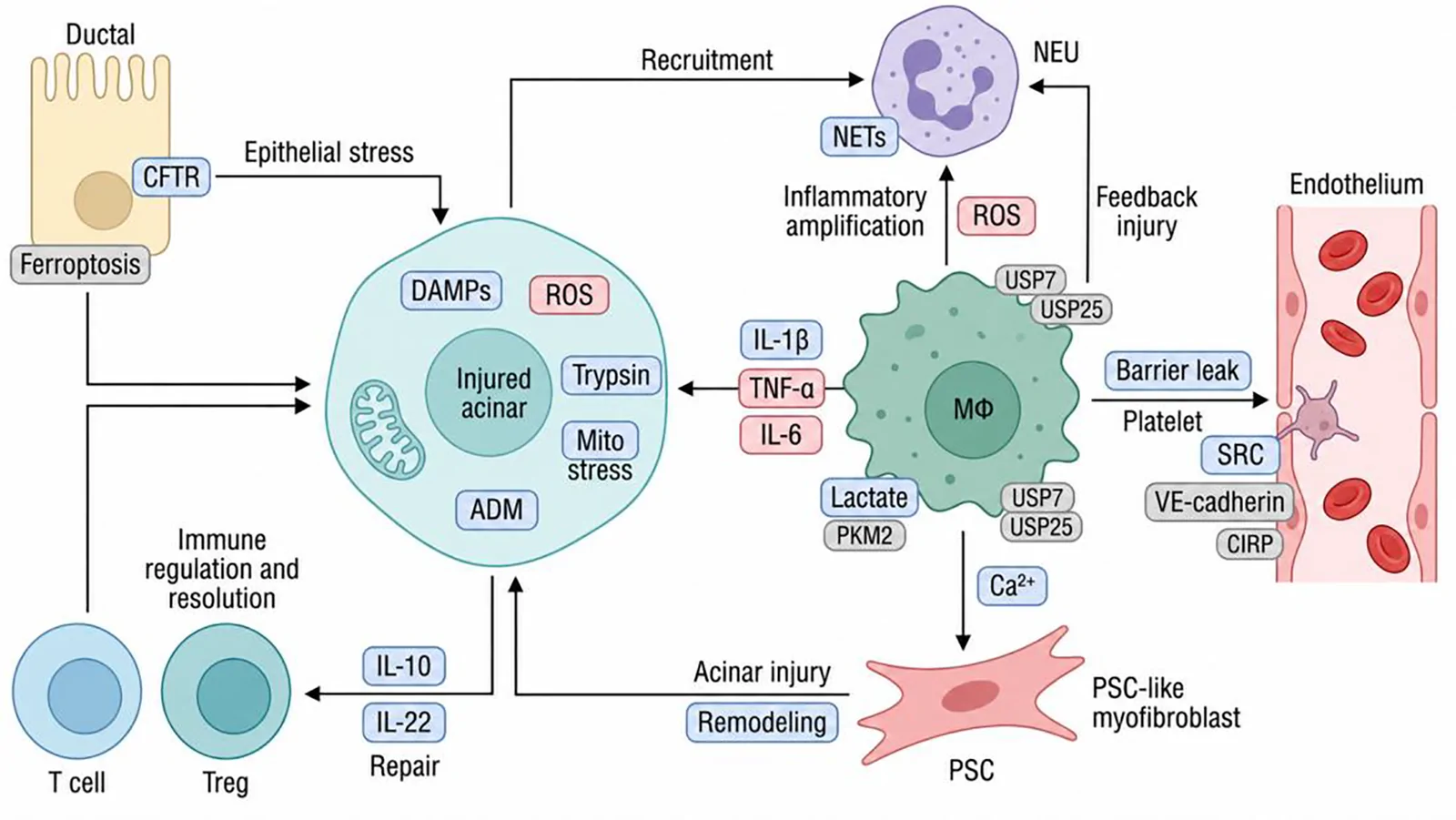
PTM-governed intercellular networks driving innate immune amplification and inflammatory resolution in acute pancreatitis. Acinar cell injury initiates a multicellular signaling program in which epithelial, immune, stromal, and vascular compartments interact to shape disease progression. Damage-associated molecular patterns (DAMPs), ROS, trypsin-associated stress, and metabolic cues derived from injured acinar cells promote macrophage activation, neutrophil recruitment, ductal stress, pancreatic stellate cell remodeling, and endothelial barrier dysfunction, while regulatory T-cell-associated signals contribute to resolution. These interconnected pathways position the pancreatic microenvironment as a dynamic decision network that governs the balance between persistent inflammation, vascular injury, and tissue repair. ADM, acinar-to-ductal metaplasia; DAMPs, damage-associated molecular patterns; IL, interleukin; MΦ, macrophage; NETs, neutrophil extracellular traps; PSC, pancreatic stellate cell; ROS, reactive oxygen species; TNF-α, tumor necrosis factor-α. Cell-type attribution is considered direct only when supported by cell-specific or cell-resolved evidence; other links represent compartment-level associations summarized with evidence qualifiers in the tables.

**Table 2:** Cell-specific and compartment-associated PTM-linked inflammatory circuits in acute pancreatitis.

Cell type/compartment	PTM-linked mechanism or pathway	Immune-inflammatory consequence	Potential translational relevance	Evidence basis	Reference
Acinar cells	EP300–HSF1–PRKN; PINK1/PARK2; lactylation-associated stress signaling	Initiate AP through organelle stress, enzyme activation, mitochondrial dysfunction, and DAMP release	Candidate preservation of mitophagy and limitation of early injury	Direct cell-specific/resolved for EP300–HSF1; AP tissue evidence for some other axes	(15, 17, 41)
Ductal cells	Ferroptosis-related lipid stress; MGST1–ACSL4/GPX4 axis	Contribute to ductal defense failure and epithelial injury amplification	Potential protection of ductal integrity; requires PTM-specific validation	AP tissue/systemic evidence—cell attribution indirect; non-AP/contextual for some mechanisms	(73, 75, 77)
Macrophages	USP7–PKM2; USP25–TBK1/NF-κB; lactate/lactylation signaling	Drive inflammatory amplification or reparative polarization depending on stage	Direction- and stage-specific macrophage reprogramming	Direct cell-specific/resolved for USP7 and recovery-stage lactylation; pathway-level for USP25	(16, 18, 19)
Neutrophils	ROS restriction; NET-associated inflammatory signaling	Amplify pancreatic injury, macrophage recruitment, and extra-pancreatic organ damage	Candidate NET/ROS control; not a direct PTM target	Direct cell-specific/resolved pathway evidence; PTM event not directly shown	(83, 84, 100)
T cells/Tregs	IL-22-related regulation; IL-10-producing Treg-associated immune control	Influence resolution versus persistent inflammation	Potential support of protective immune regulation	AP-associated immune evidence—PTM attribution indirect	(85, 86, 89)
Pancreatic stellate cells	Stress-responsive Ca²^+^ signaling; remodeling-associated stromal activation	Mediate stromal interpretation of injury, vascular interaction, and tissue remodeling	Potential modulation of harmful activation while preserving protective support	AP tissue/systemic evidence—cell attribution indirect; contextual evidence included	(91, 92)
Endothelial cells	SRC–VE-cadherin/β-catenin-associated permeability signaling	Promote barrier leak, edema, and systemic injury	Potential preservation of endothelial junctions	AP tissue/systemic evidence—cell attribution indirect	(12, 93, 96)
Platelets	Trypsin–PAR4 activation; platelet–endothelial inflammatory coupling	Contribute to immunothrombosis and microcirculatory dysfunction	Potential reduction of platelet-driven vascular injury	Direct human platelet pathway evidence; not a direct PTM event	(12, 95, 97)
Other cells	Mast-cell barrier signals; endocrine–exocrine stress crosstalk	Extend AP to gut barrier dysfunction and metabolic instability	Exploratory compartment-focused strategies	Cell-resolved AP evidence for mast cells; endocrine component contextual	(98, 99)

Evidence status: A, direct AP PTM evidence (the specific PTM was measured/manipulated and functionally linked; cell attribution is direct only when cell-specific or cell-resolved); B, AP-associated pathway, omics, or enzyme evidence without a direct endogenous substrate/site or cell-specific validation; C, extrapolated/contextual evidence from non-AP settings.

## PTM crosstalk in sterile inflammation and immune-mediated tissue injury

### Acinar stress and the initiation of sterile inflammation

Premature digestive enzyme activation remains a hallmark early lesion of AP, but it is no longer persuasive to regard it as a single initiating event upstream of all other pathogenic processes. Initiation of experimental AP can occur independently of fusion between lysosomes and zymogen granules (101). Cytosolic release of cathepsin B contributes directly to acinar cell death (102), while cathepsin B-mediated activation of trypsinogen in endocytosing macrophages can increase pancreatitis severity (103). Early trypsin activation can also develop independently of autophagy in caerulein-induced pancreatitis (104). These findings support a broader model in which enzyme activation is embedded within coordinated organelle failure rather than confined to a single lysosomal event.

The pancreas does not fail one organelle at a time. Calcium overload, mitochondrial depolarization, ATP depletion, defective autophagic degradation, ER stress, and lysosomal dysfunction reinforce one another through a network of organelle and cellular interactions (105). Autophagy is central to this network because it determines whether damaged proteins, zymogen granules, and organelles are removed or allowed to accumulate (21–24). In hypertriglyceridemia-related AP, rapamycin restores autophagic flux and suppresses ER stress (106). PTM-dependent quality control is directly represented by EP300–HSF1–PRKN-mediated mitophagy and by PINK1/PARK2-dependent suppression of NLRP3 activation (17, 41).

## Sterile inflammation and cytokine amplification

Once acinar injury exceeds the threshold of local containment, AP shifts toward a sterile inflammatory cascade initiated by danger signals. Intracellular HMGB1 can limit inflammatory nucleosome release, whereas extracellular HMGB1 signaling promotes inflammation; accordingly, LincRNA-EPS alleviates severe AP by suppressing HMGB1-triggered macrophage activation (107, 108). The AIM2 inflammasome correlates with inflammation and disease severity in patients with AP, and RAGE can activate AIM2 signaling in experimental disease (109, 110). These pathways illustrate how cell death releases damage-associated molecular patterns (DAMPs) that engage pattern-recognition receptors and sustain myeloid recruitment. NLRP3 inflammasome activation provides another major amplification platform linking mitochondrial stress, DAMP signaling, and inflammatory cytokine maturation (80).

Cytokine amplification extends local injury into a systemic process. Early trajectories of IL-1β, IL-6, IL-8, IL-10, IL-12, IFN-γ, and TNF-α vary according to disease severity (111). The measured cytokine trajectories support IL-6 as a prominent marker of severe inflammatory activation (111). PTMs help explain why this inflammatory program persists. USP25 restrains macrophage TBK1–NF-κB signaling, and its loss intensifies pancreatic and lung injury (19), whereas USP7 promotes a pro-inflammatory macrophage state through PKM2 deubiquitination (18). Thus, the cytokine response reflects a post-translationally organized amplification circuit rather than a simple consequence of necrosis.

## Mitochondrial dysfunction as an inflammatory signaling hub

Mitochondrial failure marks a critical point at which AP becomes increasingly difficult to reverse. Vitamin B12 can attenuate experimental AP by improving mitochondrial dysfunction through a CBS/SIRT1-dependent mechanism (112). Conversely, mitochondrial DNA leakage promotes persistent acinar-cell injury through cGAS–STING–NF-κB signaling (113), while CIRP blockade improves mitochondrial function, restores autophagic signaling, and suppresses pyroptosis (114). Circulating mitochondrial DNA also has potential as a biomarker of AP severity (115). These findings support a central role for mitochondrial DAMPs in converting bioenergetic failure into sterile inflammation (113–115).

Mitochondrial fate in AP is determined by competing stress-adaptive and destructive programs. Sestrin2 balances mitophagy and apoptosis through the PINK1– Parkin pathway (116), saikosaponin D limits mitochondrial damage and cGAS– STING-associated pyroptosis (117), and resveratrol promotes mitochondrial biogenesis through SIRT1/PGC-1α signaling (118). PTM-linked mechanisms of differing specificity converge on the same decision: EP300-dependent acetylation stabilizes HSF1 and preserves PRKN-mediated mitophagy (17), SIRT4 restrains HIF-1α/HO-1-associated ferroptosis (42), and PINK1/PARK2-dependent quality control suppresses NLRP3 activation (41).

This framework helps explain why oxidative stress in AP is difficult to treat once established. ROS feed back into lysosomal dysfunction, mitophagy failure, lipid peroxidation, and cytokine amplification; the resulting network helps explain the context-dependent effects of antioxidant strategies. Mitochondrial dysfunction therefore occupies a genuine pathological crossroads, linking metabolic collapse to sterile inflammation through a PTM-governed network rather than through a single isolated pathway.

## Inflammatory cell death and immune amplification

### Apoptosis

In AP, apoptosis is often regarded as less disruptive than membrane-rupturing necrosis because caspase-dependent cell clearance can limit uncontrolled release of intracellular contents. Nevertheless, apoptosis is not uniformly protective. EMC6– APAF1 signaling can promote acinar apoptosis together with inflammatory injury (119), whereas venetoclax protects against experimental AP by shifting acinar-cell death away from necrosis and toward apoptosis (120). The relevant issue is therefore one of balance: restrained and efficiently cleared apoptosis may limit tissue damage, whereas excessive apoptosis in a pancreas unable to restore proteostasis may still contribute to parenchymal loss.

### Pyroptosis

Pyroptosis transforms stressed acinar and immune cells into amplifiers of inflammation by coupling membrane-pore formation with release of IL-1β, IL-18, and other danger signals. The NLRP3–caspase-1–GSDMD axis is directly activated in acinar cells during AP and contributes to systemic inflammation (121). Downregulating or therapeutically targeting GSDMD reduces experimental severe AP and related lung injury (122, 123), while IL-37 and HDL suppress inflammasome-associated acinar-cell pyroptosis through distinct mechanisms (124, 125). GSDMD-mediated pyroptosis can exert opposing effects by resisting enzymatic digestion while expanding inflammation (126), and human placental mesenchymal stem cells attenuate SAP by inhibiting NLRP3-mediated acinar-cell pyroptosis (127). Integrated cell-death signaling is also relevant: ZBP1–PANoptosome-mediated PANoptosis contributes to AP, and eriodictyol reduces inflammation and necrosis through ZBP1-dependent signaling (128, 129).

### Ferroptosis

Ferroptosis is a credible mechanism of AP progression. Wedelolactone alleviates AP and associated lung injury through GPX4-linked suppression of pyroptosis and ferroptosis (130), while AP-1-mediated transcriptional suppression of GPX4 promotes acinar-cell ferroptosis (131). Ferroptosis also exacerbates hyperlipidemic AP by increasing lipid peroxidation and remodeling the immune microenvironment (132), whereas 4-octyl itaconate suppresses ferroptotic pancreatic and lung injury (133). PTM-focused studies place ferroptosis at the intersection of metabolism and protein regulation: lactate-driven H3K18 lactylation increases NCOA4 transcription (15), OGT-mediated O-GlcNAcylation of ATF3 activates the ACSL1 ferroptotic program (65), and SIRT4 limits ferroptosis through the HIF-1α/HO-1 axis (42).

### Other cell death programs

Necroptosis remains the clearest additional cell-death program in AP. Experimental studies have reported injurious RIPK1/RIPK3/MLKL signaling, a protective role for necroptosis in selected settings, macrophage-polarizing effects of MLKL, and clinically detectable circulating RIPK3 and MLKL, underscoring pronounced model and compartment dependence (49–53). Reviews and phosphorylated-MLKL-targeting studies likewise caution against indiscriminate inhibition of this pathway (63). AP therefore does not follow a single dominant cell-death program. Instead, multiple pathways overlap and share molecular machinery, as illustrated by ZBP1-dependent PANoptosis (128, 129). The key question is which combination of death programs predominates in a given cell type and disease stage.

## Immunovascular dysfunction and barrier failure

Barrier failure is an early and generalized component of AP rather than only a late consequence of organ failure (134). Obesity further exacerbates early gastrointestinal injury and intestinal-barrier dysfunction (135). TLR5-dependent intestinal bacterial translocation contributes to systemic inflammatory propagation after severe AP (136). In parallel, endothelial leakage is driven by CIRP–SRC signaling and disruption of VE-cadherin/β-catenin junctions (12), while glycocalyx injury contributes to severe AP-associated microvascular failure (96).

Immunovascular and barrier injury also contributes to distant-organ dysfunction. Acute kidney injury is a recognized complication of AP, with network-level inflammatory mechanisms and identifiable clinical risk factors (137, 138). Experimental certolizumab treatment reduces pancreatic and target-organ injury, supporting the feasibility of interrupting systemic inflammatory propagation (139). Platelet activation adds a further immunothrombotic layer, because pancreatic trypsin activates human platelets through PAR4 (97). Once these loops are established, the microcirculation and distant barriers become active drivers rather than passive targets of inflammation.

## Systemic inflammation, immune resolution, and disease progression

Persistent organ failure is the principal determinant of severe AP and reflects the convergence of inflammatory, metabolic, and vascular disturbances (1, 4). Lung injury models demonstrate extensive immune and tissue heterogeneity (140), while the combination of intestinal-barrier damage, systemic inflammatory response syndrome, and acute lung injury forms a mutually reinforcing axis (141). Recovery capacity is also age dependent: impaired pancreatic regeneration in older animals is associated with an exaggerated pro-inflammatory response (142). PRRX1 regulates acinar-toductal metaplasia and related plasticity (143), while clinical studies confirm an association between severe AP and new-onset diabetes after recovery (144, 145).

Repair reflects reprogramming of inflammation rather than its complete absence. Lactate-driven lactylation can promote reparative macrophage polarization and pancreatic regeneration (16), while acinar cells enter metaplastic states that may preserve viability and support subsequent redifferentiation (9, 10). However, progression from an initial episode of AP to recurrent AP and then chronic pancreatitis is a genuine clinical trajectory, particularly in alcohol-related disease (146, 147). Prospective human work has further identified distinct cytokine signatures and trajectories in severe AP (148), while angiopoietin-2 serves as an early indicator of pancreatic–renal dysfunction (149). PTM remodeling may therefore influence whether the pancreas returns to baseline, remains trapped in maladaptive repair, or progresses toward chronic fibroinflammatory remodeling (**Table 3**).

**Table 3:** PTM-linked inflammatory events and evidence-calibrated therapeutic implications in acute pancreatitis.

Pathological event	PTM-linked mechanism or downstream pathway	Immune-inflammatory consequence	Potential relevance	PTM specificity/evidence status	Reference
Digestive enzyme activation and organelle stress	Phosphorylation-dependent stress signaling; EP300–HSF1–PRKN; ubiquitin-dependent quality control	Couples calcium toxicity, lysosomal dysfunction, and impaired mitophagy to early acinar injury	Candidate early organelle-quality-control strategy	A for EP300-dependent HSF1 acetylation; otherwise B/pathway-level	(17, 101, 106)
Sterile inflammation and cytokine amplification	USP25–TBK1–NF-κB; USP7–PKM2; downstream NLRP3 signaling	Drives DAMP-triggered cytokine production, inflammasome activation, and myeloid recruitment	Potential relevance of upstream inflammatory hubs	A for USP7–PKM2; B for USP25 and NLRP3 pathway regulation	(18, 19, 107)
Mitochondrial dysfunction and oxidative stress	EP300–HSF1–PRKN; SIRT1–PINK1; SIRT4–HIF-1α/HO-1; PINK1/PARK2	Determines whether damaged mitochondria are cleared or persist as inflammatory ROS sources	Candidate mitophagy/stress-adaptation strategy	A for EP300–HSF1; B for enzyme/pathway-associated mechanisms without defined substrates	(17, 41)
Apoptosis	EMC6–APAF1; stress-associated phosphorylation signaling	Shapes acinar cell loss and may limit or aggravate injury depending on context	Potential shift away from destructive membrane-rupturing death; requires validation	B; apoptosis nodes are downstream/pathway-level, not PTMs	(119, 120)
Pyroptosis	Downstream NLRP3–caspase-1–GSDMD; CIRP-related mitochondrial stress	Links mitochondrial dysfunction to inflammatory cell death and cytokine release	Candidate context-specific control of pyroptotic amplification	B; NLRP3, GSDMD, and CIRP are pathways/molecules, not PTMs	(114, 121, 126)
Ferroptosis	H3K18la–NCOA4; OGT–ATF3–ACSL1; SIRT4–HIF-1α/HO-1	Converts metabolic and lipid stress into iron-dependent oxidative injury	Candidate identification of a ferroptosis-prone state	A for H3K18la and ATF3 O-GlcNAcylation; B for SIRT4 pathway	(15, 42, 65)
Other cell-death programs	Downstream RIPK3/MLKL and ZBP1–PANoptosome pathways	Contribute to context-dependent necroptosis and PANoptosis	Stage- and model-specific candidate pathways	B; downstream regulated-death pathways, not direct PTM crosstalk	(51, 128, 129)
Microvascular dysfunction and barrier failure	Downstream SRC–VE-cadherin/β-catenin and trypsin–PAR4 activation	Promotes endothelial leak, edema, leukocyte adhesion, and microcirculatory failure	Potential barrier- and platelet-focused strategy	B; CIRP/PAR4/barrier mechanisms are PTM-adjacent pathways	(12, 96, 97)
Systemic complications, repair, and progression	Lactate/lactylation-driven macrophage reprogramming; downstream ADM-associated plasticity	Influences organ failure, recovery, maladaptive repair, or chronic progression	Stage-specific endotyping and intervention require validation	A for recovery-stage macrophage mechanism; C for ADM contextual inference	(16, 143)

Evidence status: A, direct AP PTM evidence (the specific PTM was measured/manipulated and functionally linked; cell attribution is direct only when cell-specific or cell-resolved); B, AP-associated pathway, omics, or enzyme evidence without a direct endogenous substrate/site or cell-specific validation; C, extrapolated/contextual evidence from non-AP settings.

## Therapeutic opportunities targeting PTM-regulated inflammation in acute pancreatitis

To avoid conflating PTM-directed intervention with general AP therapy, we distinguish PTM-linked mechanistic targets from enabling strategies that inform therapeutic timing, risk stratification, or drug delivery but are not themselves PTM-targeted therapies or PTM companion diagnostics.

## Targeting PTM enzymes

The strongest therapeutic argument is no longer simply that PTMs are relevant to AP, but that selected PTM enzymes lie close to critical disease bottlenecks. Calcium-linked intervention points remain important candidates for early mechanism-based therapy. EP300-dependent acetylation preserves HSF1–PRKN-mediated mitophagy (17), whereas USP7 promotes inflammatory macrophage reprogramming through PKM2 and aggravates acinar injury through ATF4-associated autophagy (18, 55). OGT promotes NF-κB activation and ATF3–ACSL1-associated ferroptosis (64, 65), while HDAC-directed interventions have reduced enzyme activation, leukocyte recruitment, edema, and inflammatory injury in experimental AP (43–46). In contrast to USP7, USP25 restrains macrophage TBK1–NF-κB signaling, and its loss exacerbates pancreatic and lung injury (19). FGF21- and APOA1-mRNA-based therapies provide an additional translational example of reinforcing protective metabolic programs (150). Thus, PTM-enzyme targeting should be cell-specific, direction-specific, and stage-specific rather than based on class-wide inhibition.

## Targeting upstream metabolism and stress signaling

An equally important strategy is to intercept the metabolic and stress conditions that drive PTM remodeling. AMPK activation suppresses acinar-cell necroptosis and restores autophagy through SIRT1 (48, 62), FGF21 activates the SIRT1–autophagy pathway (40), betulinic acid promotes SIRT1–PINK1-mediated mitophagy (47), and CIRP blockade improves mitochondrial function and suppresses pyroptosis (114). Translation will nevertheless depend on therapeutic timing and trial design. Current clinical development includes a randomized pilot protocol for pirfenidone (151), analyses of the early therapeutic window in predicted severe AP (152), and randomized trials of sequential parecoxib–imrecoxib treatment and the calcium-release-activated calcium-channel inhibitor zegocractin (153, 154). These studies define clinical feasibility and therapeutic timing in AP rather than validating pirfenidone, parecoxib–imrecoxib, or zegocractin as PTM-directed drugs; they reinforce the need to restore adaptive stress responses before the injury network becomes biologically entrenched.

## Targeting cell-specific pathogenic circuits

Cell-specific delivery offers a practical route to precision intervention. Lure- and-kill macrophage nanoparticles reduce experimental AP severity (155), while macrophage-mimicking nanotherapy accumulates in inflamed pancreas and lung and attenuates systemic inflammation (156). Tuftsin nanoparticles target P2X7-mediated mitochondrial ROS and mitophagy (157), and an inflammation-driven nanopolyphenol system suppresses macrophage PANoptosis and pancreatic-enzyme oversecretion (158). Biomimetic ulinastatin nanoparticles provide targeted anti-inflammatory delivery (159), neutrophil-membrane-coated nanoparticles deliver celastrol to inflamed tissue (160), and ROS-scavenging nanosystems enhance mitochondrial autophagy (161). These approaches complement cell-intrinsic targets such as EP300–HSF1–PRKN, USP7–PKM2, USP25–TBK1–NF-κB, neutrophil ROS/NET pathways, and endothelial barrier signaling (12, 17–19, 84).

## Biomarkers and translational applications

Biomarker research in AP becomes more informative when it reflects underlying mechanisms instead of disease severity alone. Serum angiopoietin-2 predicts persistent organ failure, with meta-analytic evidence supporting its prognostic accuracy (162, 163). A multicenter study identified a three-miRNA serum exosomal signature that predicted persistent organ failure (164). Commentary on prognostic-biomarker reproducibility has emphasized the need for robust, externally validated assays (165), and a five-cytokine panel outperformed several conventional predictors (166). Broader biomarker reviews support the use of mechanism-informed panels rather than isolated analytes (167), while activin A independently predicted severe disease (168). Machine-learning approaches—including random forest models, EASY-APP, and broader AI frameworks—have further improved early severity prediction (169–172), while a liquid neural-network model represents a newer dynamic prediction strategy (173). Peripheral single-cell profiling adds a complementary biological layer through a B-cell/plasma-cell-associated severity signature (88). These biomarkers and prediction models currently stratify disease severity or biological state rather than directly measuring PTM status and should not be described as validated PTM companion diagnostics.

## Other emerging strategies

Exosome- and extracellular-vesicle-based strategies may provide both disease reporters and therapeutic vehicles. AP generates at least two biologically distinct exosome populations (174), and mechanistic reviews link exosomes to cell-death regulation and clinical translation (175). Preclinical evidence for stem-cell-derived extracellular vesicles has been synthesized in a systematic review and meta-analysis (176), while mesenchymal-stem-cell-derived nanovesicles have undergone direct experimental validation in AP (177). Exosomal microRNAs offer additional diagnostic and therapeutic opportunities. At present, these approaches remain preclinical, but they may provide spatial, temporal, and compartment-specific delivery precision for future interventions whose payload, target, or patient-selection rule is explicitly PTM-defined (**Figure 3**). Likewise, exosomal and nanotechnology platforms are enabling delivery technologies; their inclusion here reflects potential spatial and temporal delivery precision rather than evidence that the platforms themselves are PTM-specific therapies.

**Figure 3:**
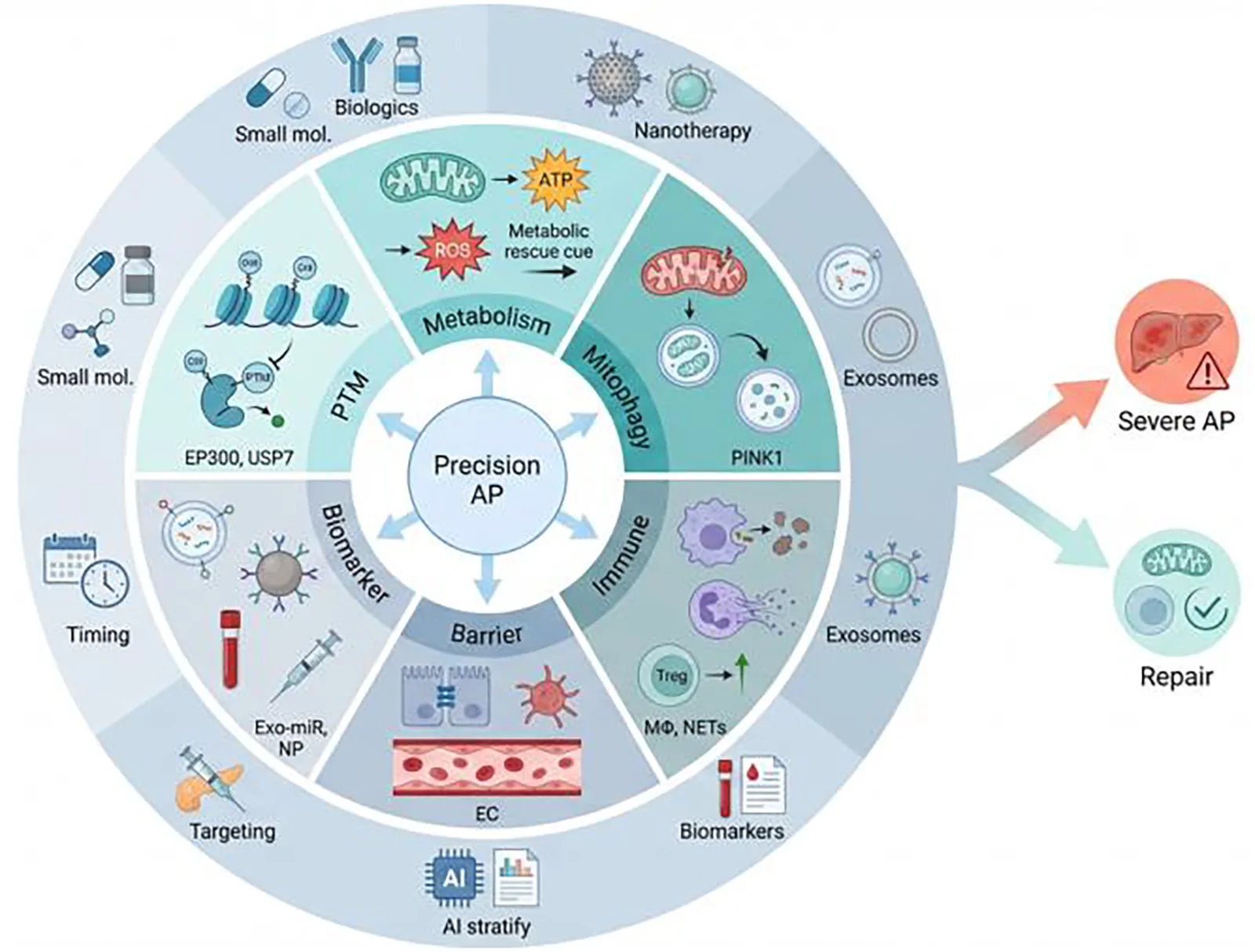
PTM-linked precision-enabling strategies for controlling inflammation in acute pancreatitis. Candidate strategies integrate six dimensions—PTM targeting, metabolic rescue, mitophagy restoration, immune reprogramming, barrier protection, and biomarker/delivery-guided stratification—together with translational tools such as small molecules, biologics, nanotherapy, exosomes, AI-assisted stratification, and targeted intervention. This framework emphasizes that stage-specific and cell-specific therapy may shift AP from severe inflammatory progression toward repair. AI models, exosomes, and nanotherapies are shown as enabling platforms for stratification or delivery and should not be interpreted as validated PTM-specific diagnostics or therapies. The schematic does not assign equal evidentiary weight to all nodes.

## Discussion

Overall, the evidence is best organized as a four-step sequence: acinar metabolic and organelle stress → PTM remodeling → innate immune and vascular amplification → inflammatory resolution or maladaptive repair. Importantly, most AP studies establish individual PTM-regulated axes rather than strict molecular PTM–PTM crosstalk. H3K18la–NCOA4, EP300–HSF1–PRKN, USP7–PKM2, and USP25–TBK1–NF-κB are therefore treated here as AP-specific PTM-linked mechanisms of differing evidentiary strength, not as proof that distinct PTMs directly modify one another. Direct molecular crosstalk in the strict biochemical sense remains insufficiently demonstrated in AP. Apparently divergent findings involving lactylation, RIPK3–MLKL, GSDMD, and USP25 further indicate that PTM effects depend on cell identity, disease stage, and experimental model. This distinction supports temporally and spatially resolved inflammatory endotyping while preventing whole-tissue, circulating-biomarker, or pathway-level associations from being overinterpreted as cell-specific PTM events.

### Current challenges

The central challenge in this field lies in placing candidate mechanisms in the correct biological sequence. Experimental AP models differ substantially in etiology, severity, and the components of human disease that they reproduce (178–180). This makes PTM biology difficult to interpret because a modification that preserves mitophagy or restrains inflammation in one model or cell type may amplify ferroptosis, cytokine signaling, or maladaptive repair in another. Proteomic studies have expanded mechanistic coverage in AP (181), but analysis of PTMs in human biopsies remains limited by sensitivity, sample input, and tissue heterogeneity (182). Low-input sequential enrichment strategies are beginning to address these constraints by enabling multi-PTM profiling from microgram-scale samples (183). A further limitation is evidence granularity: whole-pancreas acetylomic or phosphoproteomic datasets can establish tissue-level PTM remodeling but cannot, without cell-resolved validation, assign a PTM event to acinar, ductal, immune, stromal, or endothelial compartments.

Technical standardization remains a further challenge. Integrated workflows such as MoSAIC aim to improve confident analysis of PTM landscapes (184), but translation still requires alignment with emerging therapeutic mechanisms (185) and evidence-based personalized care (186). Clinical heterogeneity is increasingly being formalized through dynamic vital-sign subphenotypes, calcium-trajectory subphenotypes, and severe-AP clinical subtypes with distinct associations with early management (187–189). For PTM-directed therapy, identifying a modifier is therefore insufficient; the field must determine when to intervene, in which cell population, and whether the target functions as a pathogenic amplifier or as part of an adaptive recovery program.

### Future perspectives

The next major advance will come from time-resolved and cell-resolved integration of molecular data rather than from identifying additional PTMs in isolation. Multi-omics analyses are beginning to connect pancreatitis genetics with disease progression (190), while metabolomic liquid biopsy improves early warning of severe AP (191) and lipid-metabolic profiling supports diagnostic modeling in hyperlipidemic AP (192). Together with pancreas-focused transcriptomic and spatial studies, these approaches should clarify where, when, and in which cell types PTM remodeling becomes maladaptive.

A second priority is molecular endotyping and reproducible prediction. Federated learning has enabled multicenter early-severity prediction (193), and contemporary AI work includes dedicated reviews, supervised machine-learning models, meta-analyses, and reporting-quality assessments (194–197). These tools should ultimately integrate molecular, imaging, and clinical data into actionable endotypes. The most useful biomarker will probably not merely predict severity, but indicate whether mitophagy failure, ferroptosis-prone metabolism, endothelial collapse, or myeloid inflammatory amplification is the dominant PTM-linked or downstream circuit.

The third frontier is cell-resolved validation and longitudinal translation. A single-cell atlas of human pancreatic islet and acinar endothelial cells provides a framework for resolving vascular and endocrine compartments (198), while newly defined acinar metaplastic states refine the interpretation of exocrine-cell plasticity (199). Longitudinal symptom-network trajectories may add a patient-centered dimension to molecular and clinical endotyping (200). These advances should be integrated with high-sensitivity PTM proteomics and targeted delivery platforms to enable intervention before AP becomes biologically entrenched (**Table 4**).

**Table 4:** Stage-, cell-, and evidence-resolved translational framework for PTM-linked mechanisms in acute pancreatitis.

Disease stage/therapeutic window	Dominant compartment	PTM-linked candidate	Potential translational role	Evidence status and key limitation	Reference
Early injury/predicted severe AP, before established organ failure	Acinar cells	EP300–HSF1–PRKN; PINK1/PARK2; AMPK–SIRT1	Preserve mitophagy/organelle quality control; candidate early mechanism-directed intervention	Primarily preclinical AP evidence; PTM specificity varies; no validated human PTM companion assay	(17, 41, 48, 62)
Early ferroptosis-prone state	Acinar cells	H3K18la–NCOA4; OGT–ATF3–ACSL1; SIRT4–HIF-1α/HO-1	Identify/interrupt a ferroptosis-prone metabolic state	H3K18la and ATF3 O-GlcNAcylation are direct experimental PTM evidence; SIRT4 is PTM-associated unless substrate deacetylation is shown; human validation lacking	(15, 42, 65)
Innate immune amplification	Macrophages	USP7–PKM2; USP25–TBK1–NF-κB	Direction-specific macrophage reprogramming	AP macrophage evidence; USP25 is a protective restraint and not a simple inhibition target; direct substrate relationship must be distinguished from pathway regulation	(18, 19)
Neutrophil/immunovascular amplification	Neutrophils, endothelium, platelets	ROS/NET restriction; CIRP–SRC–VE-cadherin; trypsin–PAR4	Compartment-focused control of systemic amplification/barrier failure	Downstream/pathway-level mechanisms, not direct PTM crosstalk	(12, 84, 97)
Resolution/repair	Macrophages	Lactate/lactylation-associated reparative polarization	Support recovery-stage inflammatory resolution	Strong stage dependence; argues against blanket lactylation inhibition	(16)
Risk stratification	Circulation/PBMCs	Angiopoietin-2; exosomal miRNAs; PBMC immune signature	Early severity/biological-state stratification	Not validated PTM-specific biomarkers or PTM companion diagnostics	(88, 162, 164)
Delivery platforms	Inflamed pancreas/myeloid compartment	Biomimetic nanoparticles; extracellular vesicles; targeted nanotherapy	Spatial/temporal delivery platform	Only PTM-guided when the payload, molecular target, or patient selection is explicitly PTM-defined	(155, 159, 161, 176, 177)

Evidence status: A, direct AP PTM evidence (the specific PTM was measured/manipulated and functionally linked; cell attribution is direct only when cell-specific or cell-resolved); B, AP-associated pathway, omics, or enzyme evidence without a direct endogenous substrate/site or cell-specific validation; C, extrapolated/contextual evidence from non-AP settings.

## Conclusion

PTM remodeling and functional convergence provide a mechanistic framework for understanding how acinar metabolic stress evolves into sterile inflammation and repair, while direct molecular PTM–PTM crosstalk remains an important but incompletely validated layer of AP biology. Rather than acting uniformly, PTM pathways exhibit marked cell- and stage-specific effects, with selected circuits promoting early tissue injury while others support inflammatory resolution and repair. Current evidence identifies macrophage reprogramming, neutrophil activation, inflammasome signaling, mitochondrial quality control, and immunovascular dysfunction as major interfaces between PTM remodeling and AP severity. However, most findings remain preclinical, and direct human multi-PTM evidence is limited. Future progress will require time-resolved and cell-resolved PTM profiling, immune-informed molecular endotyping, and targeted delivery strategies capable of suppressing pathogenic inflammatory circuits without impairing adaptive repair programs.

## Glossary

AARS1: alanyl-tRNA synthetase 1
ACSL1: acyl-CoA synthetase long-chain family member 1
ACSL4: acyl-CoA synthetase long-chain family member 4
ADM: acinar-to-ductal metaplasia
AI: artificial intelligence
AIM2: absent in melanoma 2
AKT: protein kinase B
AKT1: AKT serine/threonine kinase 1
AMP: adenosine monophosphate
AMPK: AMP-activated protein kinase
AP: acute pancreatitis
APAF1: apoptotic peptidase activating factor 1
ASC: apoptosis-associated speck-like protein containing a caspase recruitment domain
ATF3: activating transcription factor 3
ATF4: activating transcription factor 4
ATP: adenosine triphosphate
AUC: area under the curve
Ca²^+^: calcium ion
CBP: CREB-binding protein
CBS: cystathionine beta-synthase
CCL5: C-C motif chemokine ligand 5
CFTR: cystic fibrosis transmembrane conductance regulator
ChIP-qPCR: chromatin immunoprecipitation–
CIRP: cold-inducible RNA-binding protein
CUT&Tag: cleavage under targets and tagmentation
DAMPs: damage-associated molecular patterns
DNA: deoxyribonucleic acid
DUBs: deubiquitinating enzymes
EMC6: endoplasmic reticulum membrane protein complex subunit 6
EP300: E1A-binding protein p300
ER: endoplasmic reticulum
FASN: fatty acid synthase
FGF21: fibroblast growth factor 21
FTO: fat mass and obesity-associated protein
GPX4: glutathione peroxidase 4
GPR132: G protein-coupled receptor 132
GSDMD: gasdermin D
H3K18la: histone H3 lysine 18 lactylation
HDACs: histone deacetylases
HDAC5: histone deacetylase 5
HGF: hepatocyte growth factor
HIF-1α: hypoxia-inducible factor 1 alpha
HMGB1: high-mobility group box 1
HO-1: heme oxygenase 1
HSF1: heat shock factor 1
IL: interleukin
IL-22BP: interleukin-22 binding protein
JAK: Janus kinase
JAK2: Janus kinase 2
JOSD2: Josephin domain-containing protein 2
MAPK: mitogen-activated protein kinase
MGST1: microsomal glutathione S-transferase 1
miRNA: microRNA
MLKL: mixed lineage kinase domain-like protein
MODS: multiple organ dysfunction syndrome
MPO: myeloperoxidase
mPTP: mitochondrial permeability transition pore
mtDNA: mitochondrial DNA
NAD^+^: nicotinamide adenine dinucleotide
NADH: reduced nicotinamide adenine dinucleotide
NCOA4: nuclear receptor coactivator 4
NETs: neutrophil extracellular traps
NF-κB: nuclear factor kappa B
NLRP3: NOD-like receptor family pyrin domain-containing 3
O-GlcNAc: O-linked β-Nacetylglucosamine
OGA: O-GlcNAcase
OGT: O-GlcNAc transferase
P2X7: P2X purinoceptor 7
PANoptosis: coordinated inflammatory cell death integrating pyroptosis, apoptosis, and necroptosis
PAR4: protease-activated receptor 4
PARK2: parkin RBR E3 ubiquitin protein ligase, legacy symbol
PBMCs: peripheral blood mononuclear cells
PCBP2: poly(rC)-binding protein 2
PCNA: proliferating cell nuclear antigen
PI3K: phosphoinositide 3-kinase
PIAS1: protein inhibitor of activated STAT 1
PINK1: PTEN-induced kinase 1
PKM2: pyruvate kinase M2
PRKN: parkin RBR E3 ubiquitin protein ligase
PSCs: pancreatic stellate cells
PTEN: phosphatase and tensin homolog
PTMs: post-translational modifications
RIPK1: receptor-interacting serine/threonine-protein kinase 1
RIPK3: receptor-interacting serine/threonine-protein kinase 3
RNA: ribonucleic acid
RNA-seq: RNA sequencing
ROS: reactive oxygen species
SAHA: suberoylanilide hydroxamic acid
SAP: severe acute pancreatitis
scRNA-seq: single-cell RNA sequencing
SIRT1: sirtuin 1
SIRT2: sirtuin 2
SIRT3: sirtuin 3
SIRT4: sirtuin 4
SLC3A2: solute carrier family 3 member 2
SLC38A2: solute carrier family 38 member 2
SOX4: SRY-box transcription factor 4
SRC: proto-oncogene tyrosine-protein kinase Src
STAT: signal transducer and activator of transcription
STAT1: signal transducer and activator of transcription 1
STAT3: signal transducer and activator of transcription 3
ST2: interleukin-1 receptorlike 1
STING: stimulator of interferon genes
SUMO: small ubiquitin-like modifier
TBK1: TANK-binding kinase 1
TFEB: transcription factor EB
TLR4: Toll-like receptor 4
TLR9: Toll-like receptor 9
TNF-α: tumor necrosis factor alpha
Tregs: regulatory T cells
USP7: ubiquitin-specific protease 7
USP25: ubiquitin-specific protease 25
VAV2: Vav guanine nucleotide exchange factor 2
VE-cadherin: vascular endothelial cadherin
VEGF: vascular endothelial growth factor
ZBP1: Z-DNA-binding protein 1
